# Resilient Adaptive Event-Triggered Load Frequency Control of Network-Based Power Systems against Deception Attacks

**DOI:** 10.3390/s21217047

**Published:** 2021-10-24

**Authors:** Xiao Zhang, Fan Yang, Xiang Sun

**Affiliations:** College of Mechanical & Electronic Engineering, Nanjing Forestry University, Nanjing 210037, China; ZhX1@njfu.edu.cn (X.Z.); sx032812@njfu.edu.cn (X.S.)

**Keywords:** load frequency control (LFC), deception attacks, adaptive event-triggered scheme (ETS), power systems (PSs)

## Abstract

This paper investigates the problem of networked load frequency control (LFC) of power systems (PSs) against deception attacks. To lighten the load of the communication network, a new adaptive event-triggered scheme (ETS) is developed on the premise of maintaining a certain control performance of LFC systems. Compared with the existing ETSs, the proposed adaptive ETS can adjust the number of triggering packets, along with the state changes in the presence of deception attacks, which can reduce the average data-releasing rate. In addition, sufficient conditions can be derived, providing a trade-off between the limited network communication resources and the desired control performance of PSs. Finally, an application case is presented for the PSs to demonstrate the advantages of the proposed approach.

## 1. Introduction

It is known that load frequency control (LFC) is a core component of PSs, which governs the system frequency and power exchange between regions in an optimally scheduled way [1,2,3]. Proportional-integral (PI) control has been widely utilized as a common control strategy in LFC [4,5,6]. For example, the authors in [6] studied the area LFC problem using fuzzy gain scheduling with a PI controller. With the development of the PSs, control signals are transmitted via a special power channel and an networked communication infrastructure, which brings new challenges to the PI controller design of LFC. Recently, a lot of the published literature [7,8,9,10,11] has concerned networked LFC. For instance, in [8], an active interference suppression control method was proposed for interconnected LFC systems.

Since the control signals of PSs are transmitted through the communication network, the potential risks of LFC systems increases, such as cyber attacks [12,13,14,15,16,17]. Cyber-attacks have receieved great attention in recent years [1,2]. Usually, cyber-attacks discussed in the literature include denial of service (DoS) attacks and deception attacks. The deception attackers launch a attack by destroying data integrity, such as tampering or replacing transmitted data. In [18], a new control method was proposed in distributed networks subject to deception attacks. The DoS attacks prevent data transmission by occupying the shared network channel, thereby degrading the system performance. A resilient ETS was well-designed in [19] for LFC systems under DoS attacks. In [14], the event-triggered control was studied for multi-agent systems under DoS attacks.

In NCS, the time-triggered scheme (TTS) is widely used to obtain the system information through the sampler, under which a fixed sampling interval can guarantee the desired performance even if there are uncertainties, time-delays, external disturbances, etc. [20,21]. However, too many “unnecessary” sampling signals are transmitted via the network, which leads to a waste of resources [22,23]. To deal with these shortcomings, an event-triggered scheme (ETS) has been widely applied to ease the network burden in recent decades. Compared to the TTS, the sampling data packets are released only when an event generated by some elaborate condition occurs, which can effectively improve resource utilization while ensuring a satisfied system performance [24,25,26,27]. However, due to the complexity of the system and the contradiction between better system performance and a lower data transmission rate, it is usually difficult to design the threshold of event-triggered conditions in the practical application system. Therefore, some state-of-the-art ETSs have been proposed, such as memory-based ETS and adaptive ETS. The authors in [28] proposed a memory-based ETS for T-S fuzzy systems, wherein some historical triggered data were utilized in the ETS so that the control performance can be ensured. The authors in [29,30] proposed an adaptive ETS for nonlinear systems, wherein the threshold can be adjusted with the system states. However, the problem of $H_{\infty }$-based LFC for network-based PSs under deception attacks by adopting adaptive ETS has not yet been reported, which prompted this study.

In sum, the goal of this work is to design an adaptive event-triggered controller for LFC systems subject to deception attacks. Differing from the existing ETS with a preset threshold, the improved adaptive ETS can adjust the number of triggering packets along with the state changes, under which the transmission rate can be cut down while maintaining the desired frequency performance of LFC systems under deception attacks.

## 2. Problem Formulation

**Figure 1:**
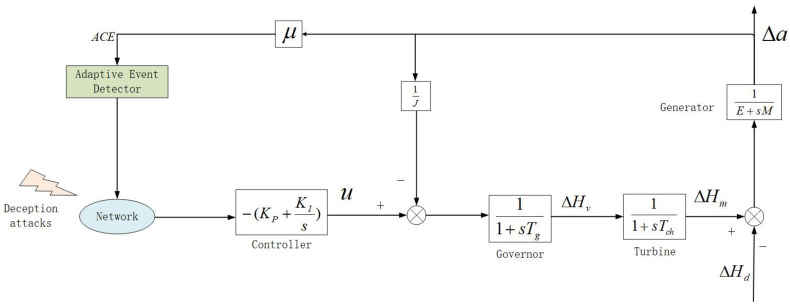
Structure of the LFC system with adaptive ETS.

Figure 1 displays a block diagram of a single-area LFC power system, where the area control error is presumed to be transmitted to the PI controller via a shared communication network.

### 2.1. Description of the LFC Systems

As shown in Figure 1, the model of the LFC systems can be indicated as follows [31]

(1) $$\begin{array}{c} \left\{\begin{array}{c} \Delta a\left(s\right)=\frac{1}{sM+E}\left(\Delta H_{m}\left(s\right)-\Delta H_{d}\left(s\right)\right),\\ \Delta H_{m}\left(s\right)=\frac{1}{1+sT_{ch}}\Delta H_{v}\left(s\right),\\ \Delta H_{v}\left(s\right)=\frac{1}{1+sT_{g}}\left(u\left(s\right)-\frac{1}{J}\Delta a\left(s\right)\right),\\ \mathrm{ACE}(s)=\mu \Delta a(s), \end{array}\right. \end{array}$$

where the symbols of the LFC system are listed in Table 1 [2].

By applying the inverse Laplace transform to (1), it can be obtained that

(2) $$\begin{array}{c} \left\{\begin{array}{c} \Delta \dot{a}\left(t\right)=\frac{1}{M}\left(\Delta H_{m}\left(t\right)-\Delta H_{d}\left(t\right)-E\Delta a\left(t\right)\right),\\ \Delta {\dot{H}}_{m}\left(t\right)=\frac{1}{T_{ch}}\left(\Delta H_{v}\left(t\right)-\Delta H_{m}\left(t\right)\right),\\ \Delta {\dot{H}}_{v}\left(t\right)=\frac{1}{T_{g}}\left(u\left(t\right)-\frac{1}{J}\Delta a\left(t\right)-\Delta H_{v}\left(t\right)\right). \end{array}\right. \end{array}$$

Similar to [10], we can obtain the state-space representation for LFC systems, as follows

(3) $$\begin{array}{c} \left\{\begin{array}{c} \dot{\hat{x}}\left(t\right)=A\hat{x}\left(t\right)+Bu\left(t\right)+F\omega \left(t\right),\\ \hat{y}\left(t\right)=C\hat{x}\left(t\right), \end{array}\right. \end{array}$$

where $\hat{x}\left(t\right)={\left[\Delta a\left(t\right)\;\;\Delta H_{m}\left(t\right)\;\;\Delta H_{v}\left(t\right)\right]}^{T}$, $\omega \left(t\right)=\Delta H_{d}\left(t\right),\hat{y}\left(t\right)=\mathrm{ACE}\left(t\right)$, and

$$\begin{array}{c} \begin{array}{c} A=\left[\begin{array}{ccc} -\frac{E}{M} & \frac{1}{M} & 0\\ 0 & -\frac{1}{T_{ch}} & \frac{1}{T_{ch}}\\ -\frac{1}{JT_{g}} & 0 & -\frac{1}{T_{g}} \end{array}\right],B={\left[0\;\;0\;\;\frac{1}{T_{g}}\right]}^{T},C=\left[\begin{array}{ccc} \mu & 0 & 0 \end{array}\right],F={\left[-\frac{1}{M}\;\;0\;\;0\right]}^{T}. \end{array} \end{array}$$

### 2.2. Adaptive ETS Controller Design

Simular to [10], the PI control strategy of the LFC systems is designed as

(4) $$\begin{array}{c} u\left(t\right)=-K_{P}\mathrm{ACE}\left(t\right)-K_{I}\int_{0}^{t}\mathrm{ACE}\left(s\right)ds, \end{array}$$

where $K_{P}$ denotes proportional gain and $K_{I}$ stands for integral gain.

For convenience of obtaining the controller gains, we transform the above PI control form into the output feedback problem. Then, we redefine the output variables

$$\begin{array}{cc} y(t)= & {\left[\mathrm{ACE}\left(t\right)\;\;\int_{0}^{t}\mathrm{ACE}\left(s\right)ds\right]}^{T}. \end{array}$$

Define $K=[K_{P}\;\;K_{I}]$, and we can rewrite (4) as

(5) $$\begin{array}{cc} u(t)= & -Ky(t). \end{array}$$

However, the sampled signal of ACE(t) of networked LFC systems will only be released to the PI controller via the network when the preset condition is satisfied [20]. To adjust the number of triggering packets in the LFC system, along with the state changes under deception attacks, an improved adaptive ETS is put forward, as follows:

(6) $$\begin{array}{c} t_{k+1}h=t_{k}h+\min_{l\in N}\left\{lh|\varkappa^{T}\left(i_{l}h\right)\phi \varkappa \left(i_{l}h\right)>\sigma \left(t\right)x^{T}\left(t_{k}h\right)\phi x\left(t_{k}h\right)\right\}, \end{array}$$

where *h* is the sampling period, ϕ is a positive symmetric matrix to be designed, $t_{k}h$ is the data-releasing instant, $\varkappa \left(i_{l}h\right)=y\left(i_{l}h\right)-y\left(t_{k}h\right)$, $i_{l}h=lh+t_{k}h,l\in N,i_{l}h\in \left(t_{k}h,t_{k+1}h\right],$$\left\{t_{0},t_{1},t_{2},\ldots \right\}\subset \left\{0,1,2,\ldots \right\}$, and

(7) $$\begin{array}{c} \sigma \left(t\right)=\alpha \left(1-\frac{2}{\pi }\arctan\left(\iota ∥y(i_{l}h)∥\right)\right), \end{array}$$

wherein α∈(0,1) is the upper bound of σ(t), ι and α are given positive constants.

**Remark** **1.** *It can be seen from (7) that σ(t) can adaptively adjusted to the system states by a arctangent function, which is different from the existing ETS with a constant threshold. When the system states fluctuate, σ(t) will be adaptively adjusted to a lower value, by which more packets with the system information can be released to the controller. When the system is stable, σ(t) will be automatically adjusted to a larger value to decrease the release rate of sampled packets.*

Based on the condition (6), one can obtain that

(8) $$\begin{array}{c} y\left(t_{k}h\right)=y\left(i_{l}h\right)-\varkappa \left(i_{l}h\right). \end{array}$$

Since the communication network is vulnerable to cyber attacks, the transmission signal can be written as

(9) $$\begin{array}{c} \hat{y}\left(t\right)=\varphi \left(t\right)ð\left(t\right)+\left(1-\varphi \left(t\right)\right)y\left(t_{k}h\right), \end{array}$$

where the Bernoulli variable φ(t)∈{0,1} is introduced to characterize the behavior of random deception attacks, $E\left\{\varphi \left(t\right)\right\}=\bar{\varphi }$, $E\left\{{\left(\varphi \left(t\right)-\bar{\varphi }\right)}^{2}\right\}=\rho^{2}$ and the nonlinear attack signal ð(t) satisfies

(10) $$\begin{array}{c} \begin{array}{c} {∥ð\left(t\right)∥}^{2}\leq {∥Gy\left(t\right)∥}^{2}, \end{array} \end{array}$$

where G is a known matrix with appropriate dimension.

**Remark** **2.** *Cyber-attack has receieved great attention in recent years since it is one of the major threats to system stability [1,2,19]. Usually, cyber-attacks discussed in the literature include DoS attacks and deception attacks. The DoS attacks prevent data transmission by occupying the shared network channel, thereby degrading system performance. The deception attackers launch an attack by destroying data integrity, such as tampering with or replacing transmitted data. In this paper, we consider a kind of deception attack when investigating the LFC problem of PSs.*

**Remark** **3.** *When φ(t)=1, the true measurement data are replaced with the data of deception attacks. Otherwise, the true measurement data can be transmitted to the controller.*

Considering the deception attacks, the output of the controller in (5) can be rewritten as

(11) $$\begin{array}{cc} u(t)= & -K\hat{y}\left(t\right). \end{array}$$

### 2.3. Closed-Loop Control of LFC Systems

According to the adaptive ETS in (6), the current signal is maintained by the zero-order holder (ZOH) until the next packet is transmitted. Therefore, we need to divide the interval Π = [$\bar{m}\;\;,\;\;\bar{n}$) into ℘+1 pieces, where $\bar{m}=t_{k}h+\lambda_{t_{k}}$, $\bar{n}=t_{k+1}h+\lambda_{t_{k+1}}$. The network-induced delay at instant $t_{k}h$ is denoted by $\lambda_{t_{k}}$ and the holding interval Π can be divided into

(12) $$\begin{array}{c} \Pi =\cup_{l=0}^{℘}\Pi_{l}, \end{array}$$

where

$$\begin{array}{cc} \; & \Pi_{l}=\left[t_{k}h+lh+\vartheta ,t_{k+1}h+\left(l+1\right)h+\vartheta \right),\\ \; & \vartheta =\left\{\begin{array}{c} \lambda_{t_{k}},\;\;\;\;\;\;l=0,1,\ldots ,℘-1,\\ \lambda_{t_{k+1}},\;\;\;l=℘, \end{array}\right.\\ \; & ℘+1=t_{k+1}-t_{k}. \end{array}$$

Define

$$\begin{array}{c} \lambda \left(t\right)=t-i_{l}h, \end{array}$$

where $i_{l}h=t_{k}h+lh$, $0\leq \lambda_{t_{k}}\leq \lambda \left(t\right)\leq \lambda_{M}=\bar{\lambda }$, $\lambda_{M}=h+\max\left\{\lambda_{t_{k}}\right\}$. Then, $\hat{y}\left(t\right)$ can be represented by

(13) $$\begin{array}{c} \hat{y}\left(t\right)=\varphi \left(t\right)ð\left(t\right)+(1-\varphi \left(t\right)\left(y\left(t-\lambda \left(t\right)\right)-\varkappa \left(t-\lambda \left(t\right)\right)\right), \end{array}$$

for $t\in \Pi_{l}.$

Redefining new variables $x\left(t\right)={\left[\Delta a\left(t\right)\;\;\Delta H_{m}\left(t\right)\;\;\Delta H_{v}\left(t\right)\;\;\int_{0}^{t}\mathrm{ACE}\left(s\right)ds\right]}^{T}$. Combine (4)–(13), the LFC systems (3) with an adaptive event-triggered PI controller against deception attacks can be formulated as

(14) $$\begin{array}{c} \left\{\begin{array}{c} \begin{array}{cc} \dot{x}\left(t\right)= & Ax(t)-(1-\varphi (t))BKCx(t-\lambda (t))+(1-\varphi (t))BK\varkappa (t-\lambda (t))+F\omega (t)\\ & -BK\varphi (t)ð(t),\\ y(t)= & Cx\left(t\right),t\in \Pi_{l}. \end{array} \end{array}\right. \end{array}$$

where

$$\begin{array}{c} A=\left[\begin{array}{cccc} -\frac{E}{M} & \frac{1}{M} & 0 & 0\\ 0 & -\frac{1}{T_{ch}} & \frac{1}{T_{ch}} & 0\\ -\frac{1}{RT_{g}} & 0 & -\frac{1}{T_{g}} & 0\\ \mu & 0 & 0 & 0 \end{array}\right],B=\left[\begin{array}{c} 0\\ 0\\ \frac{1}{T_{g}}\\ 0 \end{array}\right],C=\left[\begin{array}{cccc} \mu & 0 & 0 & 0\\ 0 & 0 & 0 & 1 \end{array}\right],F=\left[\begin{array}{c} -\frac{1}{M}\\ 0\\ 0\\ 0 \end{array}\right]. \end{array}$$

The purpose of this article is to design the adaptive event-triggered PI controller subject to deception attacks, while ensuring that E{∥y(t)∥}≤E{γ∥ω(t)∥} holds with zero initial state conditions when ω(t)≠0, and the LFC system (14) could achieve stability with ω(t)=0.

## 3. Main Results

In this section, we use the Lyapunov–Krasovskii function method to derive the stability criteria of the LFC system. Then, the weight matrix of adaptive ETS and the controller gain will be calculated by LMIs. The statement of sufficient conditions for the LFC system are shown in the following.

**Theorem** **1.** *For given scalars $\bar{\lambda }>0$, $\alpha \in \left(0,1\right),\bar{\varphi }\in \left(0,1\right),\rho$, $H_{\infty }$ norm bound γ, and matrix K, the system (14) is asymptotically stable, if there exist matrices $P>0,P_{2}>0,R>0,Q>0$W>0 and a matrix U such that*

(15) $$\begin{array}{c} \left[\begin{array}{cc} R & *\\ U & R \end{array}\right]>0, \end{array}$$

(16) $$\begin{array}{c} \Xi =\left[\begin{array}{cccc} \Xi_{11} & * & * & *\\ \Xi_{21} & -\bar{\varphi }P_{2} & * & *\\ \Xi_{31} & \Xi_{32} & -(R+W) & *\\ \Xi_{41} & \Xi_{42} & 0 & -(R+W) \end{array}\right]<0, \end{array}$$

*where*

$$\begin{array}{cc} \Xi_{11}= & \left[\begin{array}{ccccc} \Psi_{11} & * & * & * & *\\ \Psi_{21} & \Psi_{22} & * & * & *\\ U & R-U & -Q-R & * & *\\ \left(1-\bar{\varphi }\right)K^{T}B^{T}P & -\alpha \phi C & 0 & -\phi +\alpha \phi & *\\ F^{T}P & 0 & 0 & 0 & -\gamma^{2}I \end{array}\right],\\ \Psi_{11}= & A^{T}P+PA+Q-R-\frac{\pi^{2}}{4}W+C^{T}C+\bar{\varphi }C^{T}G^{T}P_{2}GC,\\ \Psi_{21}= & \left(\bar{\varphi }-1\right)C^{T}K^{T}B^{T}P+R-U+\frac{\pi^{2}}{4}W,\\ \Psi_{22}= & U+U^{T}-2R-\frac{\pi^{2}}{4}W+\alpha C^{T}\phi C,\\ \Xi_{21}= & [-\bar{\varphi }K^{T}B^{T}P\;\;0\;\;0\;\;0\;\;0],\\ \Xi_{31}= & \bar{\lambda }\left(R+W\right)\Upsilon_{1},\\ \Xi_{32}= & -\bar{\lambda }\bar{\varphi }\left(R+W\right)BK,\\ \Xi_{41}= & \bar{\lambda }\rho \left(R+W\right)\Upsilon_{2},\\ \Xi_{42}= & \bar{\lambda }\rho \left(R+W\right)BK,\\ \Upsilon_{1}= & \left[\begin{array}{ccccc} A & (\bar{\varphi }-1)BKC & 0 & (1-\bar{\varphi })BK & F \end{array}\right],\\ \Upsilon_{2}= & \left[\begin{array}{ccccc} 0 & -BKC & 0 & BK & 0 \end{array}\right]. \end{array}$$

**Proof.**  Construct a Lyapunov–Krasovskii function in [31] for the system (14) as

(17) $$\begin{array}{cc} V(t)= & x^{T}\left(t\right)Px\left(t\right)+\int_{t-\bar{\lambda }}^{t}x^{T}\left(s\right)Qx\left(s\right)ds+\bar{\lambda }\int_{t-\bar{\lambda }}^{t}\int_{s}^{t}{\dot{x}}^{T}\left(v\right)R\dot{x}\left(v\right)dvds\\ & +{\bar{\lambda }}^{2}\int_{i_{l}h}^{t}{\dot{x}}^{T}\left(s\right)W\dot{x}\left(s\right)ds-\frac{\pi^{2}}{4}\int_{i_{l}h}^{t}{\left[x\left(s\right)-x\left(i_{l}h\right)\right]}^{T}W\left[x\left(s\right)-x\left(i_{l}h\right)\right]ds. \end{array}$$

Define $ϝ\left(t\right)={\bar{\lambda }}^{2}{\dot{x}}^{T}\left(t\right)R\dot{x}\left(t\right)+{\bar{\lambda }}^{2}{\dot{x}}^{T}\left(t\right)W\dot{x}\left(t\right)$; then, the following results can be derived from (17),

(18) $$\begin{array}{cc} E\{\dot{V}\left(t\right)\}= & 2x^{T}\left(t\right)P\Lambda_{1}\left(t\right)+x^{T}\left(t\right)Qx\left(t\right)-x^{T}\left(t-\bar{\lambda }\right)Qx\left(t-\bar{\lambda }\right)-\bar{\lambda }\int_{t-\bar{\lambda }}^{t}{\dot{x}}^{T}\left(s\right)R\dot{x}\left(s\right)ds\\ & -\frac{\pi^{2}}{4}{\left[x\left(t\right)-x\left(i_{l}h\right)\right]}^{T}W\left[x\left(t\right)-x\left(i_{l}h\right)\right]+E\left\{ϝ\left(t\right)\right\}, \end{array}$$

where

$$\begin{array}{cc} E\{ϝ(t)\} & ={\bar{\lambda }}^{2}\Lambda_{1}^{T}\left(t\right)\left(R+W\right)\Lambda_{1}\left(t\right)+{\bar{\lambda }}^{2}\rho^{2}\Lambda_{2}^{T}\left(t\right)\left(R+W\right)\Lambda_{2}\left(t\right),\\ \Lambda_{1}\left(t\right)= & Ax\left(t\right)-\left(1-\bar{\varphi }\right)BKCx\left(t-\lambda (t)\right)+\left(1-\bar{\varphi }\right)BK\varkappa \left(t-\lambda \left(t\right)\right)+F\omega \left(t\right)-\bar{\varphi }BKð\left(t\right),\\ \Lambda_{2}\left(t\right)= & BK\varkappa (t-\lambda (t))-BKCx(t-\lambda (t))+BKð(t). \end{array}$$

From the adaptive ETS (6), one can obtain

(19) $$\begin{array}{c} \sigma \left(t\right){\left[y\left(i_{l}h\right)-\varkappa \left(i_{l}h\right)\right]}^{T}\phi \left[y\left(i_{l}h\right)-\varkappa \left(i_{l}h\right)\right]-\varkappa {\left(i_{l}h\right)}^{T}\phi \varkappa \left(i_{l}h\right)\geq 0. \end{array}$$

According to inequality (10), it has

(20) $$\begin{array}{c} \bar{\varphi }y^{T}\left(t\right)G^{T}P_{2}Gy\left(t\right)-\bar{\varphi }{ð}^{T}\left(t\right)P_{2}ð\left(t\right)\geq 0, \end{array}$$

where $P_{2}$ is a positive symmetric matrix.

Define $\Omega \left(t\right)=\gamma^{2}\omega^{T}\left(t\right)\omega \left(t\right)-y^{T}\left(t\right)y\left(t\right)$; then, combining (15)–(20), and using Schur complement lemma and the method in [31] follows:

(21) $$\begin{array}{cc} \; & E\left\{\dot{V}\left(t\right)\right\}\leq E\left\{\Psi^{T}\left(t\right)\Xi \Psi \left(t\right)\right\}+E\left\{\Omega \left(t\right)\right\}, \end{array}$$

where

$$\Psi^{T}\left(t\right)=\left[x^{T}\left(t\right)\;\;x^{T}\left(t-\lambda \left(t\right)\right)\;\;x^{T}\left(t-\bar{\lambda }\right)\;\;\varkappa^{T}\left(t-\lambda \left(t\right)\right)\;\;\omega^{T}\left(t\right)\;\;{ð}^{T}\left(t\right)\right].$$

According to (15) and (16), we can conclude that $E\{\Psi^{T}\left(t\right)\Xi \Psi \left(t\right)\}\leq 0$, which means that

(22) $$\begin{array}{c} E\left\{\dot{V}\left(t\right)\right\}<E\left\{\Omega \left(t\right)\right\}. \end{array}$$

Taking the integration on both sides for (22) from 0 to +∞, we have

(23) $$\begin{array}{c} E\left\{V\left(+\infty \right)-V\left(0\right)\right\}<E\left\{\int_{0}^{+\infty }\Omega \left(t\right)dt\right\}. \end{array}$$

The LFC systems (14) are asymptotically stable with zero initial conditions when ω(t)=0, and E{∥y(t)∥}≤E{γ∥ω(t)∥} when ω(t)≠0. The proof is complete. □

**Theorem** **2.** *For given scalars $\bar{\lambda }>0$, $\alpha \in \left(0,1\right),\bar{\varphi }\in \left(0,1\right),\rho$, $H_{\infty }$ norm bound γ, the system (14) is asymptotically stable, if there are symmetric and positive definite matrices L, $X,\tilde{Q},\tilde{W},\tilde{R}$, matrices $\tilde{U}$ and N with appropriate dimensions, such that the following linear matrix inequalities hold:*

(24) $$\begin{array}{c} CX=LC, \end{array}$$

(25) $$\begin{array}{c} \left[\begin{array}{cc} \tilde{R} & *\\ \tilde{U} & \tilde{R} \end{array}\right]>0, \end{array}$$

(26) $$\begin{array}{c} \tilde{\Xi }=\left[\begin{array}{ccc} {\tilde{\Theta }}_{11} & * & *\\ {\tilde{\Theta }}_{21} & {\tilde{\Theta }}_{22} & *\\ {\tilde{\Theta }}_{31} & 0 & {\tilde{\Theta }}_{33} \end{array}\right]<0, \end{array}$$

*where*

$$\begin{array}{cc} {\tilde{\Theta }}_{11}= & \left[\begin{array}{cccccc} {\tilde{\Xi }}_{11} & * & * & * & * & *\\ {\tilde{\Xi }}_{21} & {\tilde{\Xi }}_{22} & * & * & * & *\\ \tilde{U} & \tilde{R}-\tilde{U} & -\tilde{Q}-\tilde{R} & * & * & *\\ \left(1-\bar{\varphi }\right)N^{T}B^{T} & -\alpha \tilde{\phi }C & 0 & -\tilde{\phi }+\alpha \tilde{\phi } & * & *\\ {\tilde{\Xi }}_{51} & 0 & 0 & 0 & -\gamma^{2}I & *\\ -\bar{\varphi }N^{T}B^{T} & 0 & 0 & 0 & 0 & -\bar{\varphi }L \end{array}\right],\\ {\tilde{\Xi }}_{11}= & XA^{T}+AX+\tilde{Q}-\tilde{R}-\frac{\pi^{2}}{4}\tilde{W},\\ {\tilde{\Xi }}_{21}= & \left(\bar{\varphi }-1\right)C^{T}N^{T}B^{T}+\tilde{R}-\tilde{U}+\frac{\pi^{2}}{4}\tilde{W},\\ {\tilde{\Xi }}_{22}= & -2\tilde{R}+\tilde{U}+{\tilde{U}}^{T}-\frac{\pi^{2}}{4}\tilde{W}+\alpha C^{T}\tilde{\phi }C,{\tilde{\Xi }}_{51}=F^{T},\\ {\tilde{\Theta }}_{21}= & \left[\begin{array}{cccccc} \bar{\lambda }\mathrm{AX} & -\bar{\lambda }\left(1-\bar{\varphi }\right)BNC & 0 & \bar{\lambda }\left(1-\bar{\varphi }\right)BN & \bar{\lambda }F & -\bar{\lambda }\bar{\varphi }BN\\ 0 & -\bar{\lambda }\rho BNC & 0 & \bar{\lambda }\rho BN & 0 & \bar{\lambda }\rho BN \end{array}\right],\\ {\tilde{\Theta }}_{22}= & \mathrm{diag}\left\{-2\zeta_{0}X+\zeta_{0}^{2}\left(R+W\right),\;-2\zeta_{1}X+\zeta_{1}^{2}\left(R+W\right)\right\},\\ {\tilde{\Theta }}_{31}= & \left[\begin{array}{cccccc} \mathrm{LC} & 0 & 0 & 0 & 0 & 0\\ \sqrt{\bar{\varphi }}\mathrm{GLC} & 0 & 0 & 0 & 0 & 0 \end{array}\right],{\tilde{\Theta }}_{33}=\mathrm{diag}\left\{-I,\;-L\right\}. \end{array}$$

*Then, the controller gain is derived by $K=NL^{-1}$.*

**Proof.**  Define $\tilde{Q}=\mathrm{XQX},KL=N,X=P^{-1},\tilde{W}=\mathrm{XWX}>0,\tilde{R}=\mathrm{XRX},\tilde{U}=\mathrm{XUX}$, appropriate dimension matrix $L=P_{2}^{-1},\tilde{\phi }=L\phi L$.

Using pre- and post-multiplying (15) with $H_{1}$ and pre- and post-multiplying (16) with $H_{2}$, one can see that (25) and (27) hold, where $H_{1}$ = diag {X,X}, $H_{2}$ = diag {X,X,X,L,I,L,${\left(R+W\right)}^{-1},{\left(R+W\right)}^{-1}\}$.

(27) $$\begin{array}{c} \Xi =\left[\begin{array}{ccc} \Theta_{11} & * & *\\ \Theta_{21} & \Theta_{22} & *\\ \Theta_{31} & 0 & \Theta_{33} \end{array}\right]<0, \end{array}$$

where

$$\begin{array}{cc} \Theta_{11}= & \left[\begin{array}{cccccc} \Xi_{11} & * & * & * & * & *\\ \Xi_{21} & \Xi_{22} & * & * & * & *\\ \tilde{U} & \tilde{R}-\tilde{U} & -\tilde{Q}-\tilde{R} & * & * & *\\ \left(1-\bar{\varphi }\right)LK^{T}B^{T} & -\alpha L\phi CX & 0 & -\tilde{\phi }+\alpha \tilde{\phi } & * & *\\ \Xi_{51} & 0 & 0 & 0 & -\gamma^{2}I & *\\ -\bar{\varphi }LK^{T}B^{T} & 0 & 0 & 0 & 0 & -\bar{\varphi }L \end{array}\right],\\ \Xi_{11}= & XA^{T}+AX+\tilde{Q}-\tilde{R}-\frac{\pi^{2}}{4}\tilde{W},\\ \Xi_{21}= & \left(\bar{\varphi }-1\right)XC^{T}K^{T}B^{T}+\tilde{R}-\tilde{U}+\frac{\pi^{2}}{4}\tilde{W},\\ \Xi_{22}= & -2\tilde{R}+\tilde{U}+{\tilde{U}}^{T}-\frac{\pi^{2}}{4}\tilde{W}+\alpha XC^{T}\phi CX,\Xi_{51}=F^{T},\\ \Theta_{21}= & \left[\begin{array}{cccccc} \bar{\lambda }\mathrm{AX} & -\bar{\lambda }\left(1-\bar{\varphi }\right)BK\mathrm{CX} & 0 & \bar{\lambda }\left(1-\bar{\varphi }\right)BKL & \bar{\lambda }F & -\bar{\lambda }\bar{\varphi }BKL\\ 0 & -\bar{\lambda }\rho BK\mathrm{CX} & 0 & \bar{\lambda }\rho BKL & 0 & \bar{\lambda }\rho BKL \end{array}\right],\\ \Theta_{22}= & \mathrm{diag}\left\{-{\left(R+W\right)}^{-1}\;\;-{\left(R+W\right)}^{-1}\right\},\Theta_{31}=\left[\begin{array}{cccccc} \mathrm{CX} & 0 & 0 & 0 & 0 & 0\\ \sqrt{\bar{\varphi }}\mathrm{GCX} & 0 & 0 & 0 & 0 & 0 \end{array}\right],\\ \Theta_{33}= & \mathrm{diag}\{-I,\;-L\}. \end{array}$$

Noting that $\left(\zeta R-P\right)R^{-1}\left(\zeta R-P\right)\geq 0$, ζ>0, it is easy to see that $-{\mathrm{PR}}^{-1}P\leq \zeta^{2}R-2\zeta P$. Define $H_{3}=\mathrm{diag}\left\{I,I,I,I,I,I,P,P,I,I\right\}$ and $H_{4}=\mathrm{diag}\left\{I,I,I,I,I,I,X,X,I,I\right\}$. By using CX and N instead of LC and KL, and pre- and post-multiplying (27) with $H_{3}$ and $H_{4}$, respectively, one can obtain that the inequality (26) holds. This ends the proof. □

To solve the problem of equality (24) in Theorem 2, we use the optimization algorithm in [32], which can be expressed as

(28) $$\begin{array}{c} \left\{\begin{array}{c} \left[\begin{array}{ccc} -\kappa I & \; & {\left(\mathrm{LC}-\mathrm{CX}\right)}^{T}\\ \left(\mathrm{LC}-\mathrm{CX}\right) & \; & -I \end{array}\right]<0,\\ \kappa \to 0, \end{array}\right. \end{array}$$

where κ>0 is a small enough constant. Furthermore, the controller gain could be calculated by (25), (26) and (28).

## 4. Simulation Examples

An application example of LFC systems in [33,34] is given to verify the efficacy of the method, whose nominal values are listed in Table 2.

Select the attack function ð(t) = −tanh $\left(Gy(t)\right)$[2] and G = diag{0.8,0.1}. The mathematic expectation of the deception attack is given as $\bar{\varphi }=0.5$. The disturbance is chosen as

$$\omega \left(t\right)=\left\{\begin{array}{ccc} \; & 0.5\cos(0.1t),\; & 15\leq t\leq 20\\ \; & 0,\; & \mathrm{otherwise}. \end{array}\right.$$

Next, two cases are utilized to manifest the proposed method for LFC systems.

**Case 1:** The impact of deception attacks is not considered in the controller design in this case. Give the parameters $\zeta_{0}=\zeta_{1}=0.01,\kappa =0.1$. Choose the adaptive law parameters α=0.8, ι=80, sampling period h=0.05, the upper bound of network-induced delay $\bar{\lambda }=0.001$, and $H_{\infty }$ performance index γ=15. Then, the controller gain and weighting matrix can be figured out by Theorem 2 as follows

$$\begin{array}{c} K=\left[\begin{array}{cc} 0.0627 & 0.2561 \end{array}\right],\phi =\left[\begin{array}{cc} 0.3654 & 0.4298\\ 0.4298 & 2.1692 \end{array}\right]. \end{array}$$

**Figure 2:**
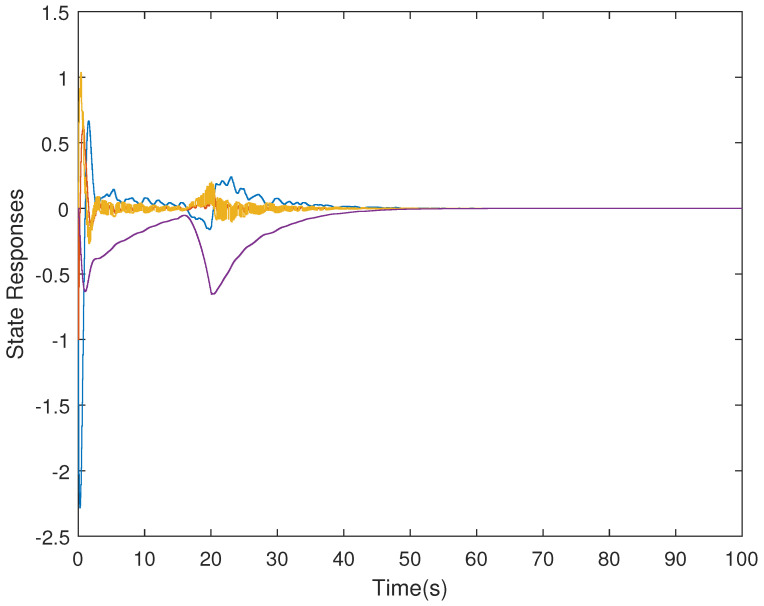
State responses of the LFC system in Case 1.

**Figure 3:**
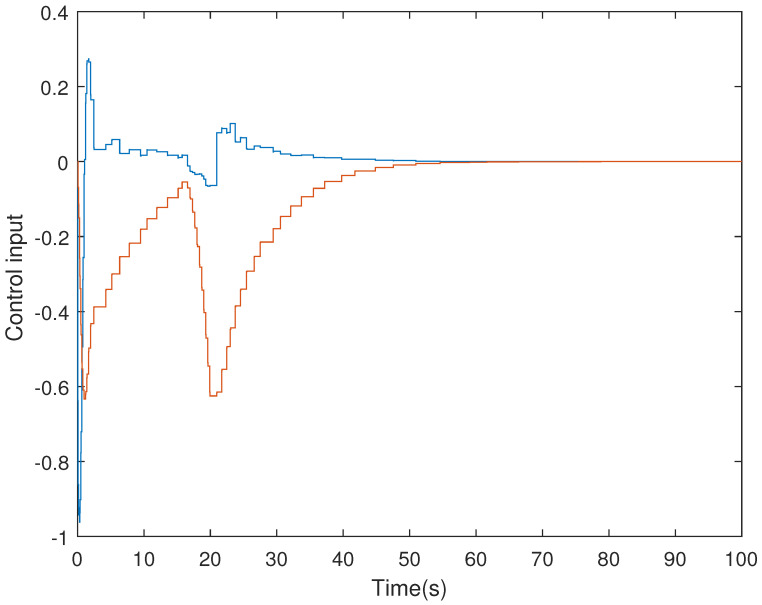
Control input of LFC systems in Case 1.

**Figure 4:**
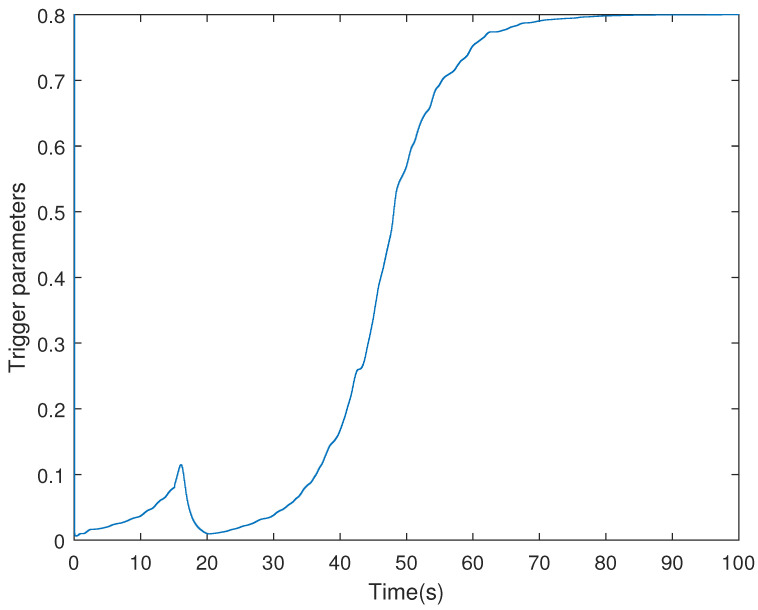
The threshold σ(t) of the LFC system with the adaptive ETS in Case 1.

**Figure 5:**
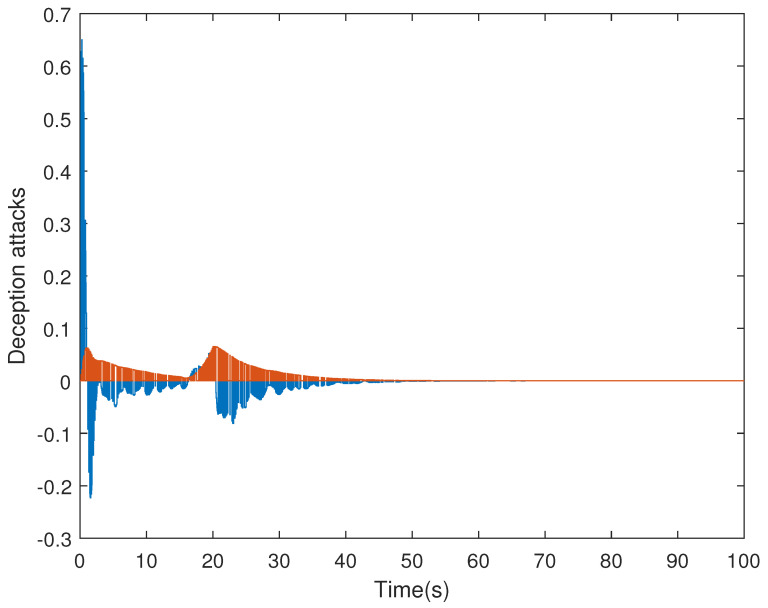
Deception attacks with $\bar{\varphi }=0.5$.

It is assumed that the initial condition of system is $x\left(0\right)={\left[-1.5\;\;-1\;\;0.2\;\;0\right]}^{T}$. The results are obtained in Figure 2, Figure 3, Figure 4 and Figure 5. The state responses of the LFC system in Case 1 are shown in Figure 2, which indicates that the LFC system is stable after 60 s. Figure 3 illustrates the responses of control input. The adaptive law σ(t) is shown in Figure 4, where the curve finally converges to the upper bound α=0.8, which indicates that the amount of transmitted signals is greatly reduced when the system is stable. Figure 5 illustrates the deception attack signals of simulation.

**Case 2:** The impact of deception attacks in the design process of the controller is considered, and the mathematic expectation of the deception attack is given as $\bar{\varphi }=0.5$. The other parameters are the same as those in Case 1. Then, we can obtain the controller gain and weighting matrix by Theorem 2 as follows

$$\begin{array}{c} K=\left[\begin{array}{cc} 0.0374 & 0.5270 \end{array}\right],\phi =\left[\begin{array}{cc} 0.2762 & 0.3004\\ 0.3004 & 4.3447 \end{array}\right]. \end{array}$$

**Figure 6:**
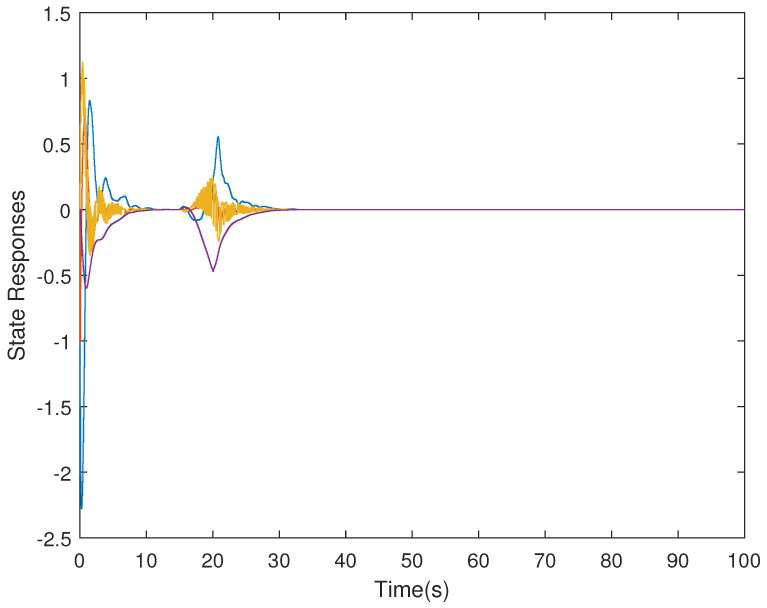
State responses of the LFC system based on the adaptive ETS in Case 2.

**Figure 7:**
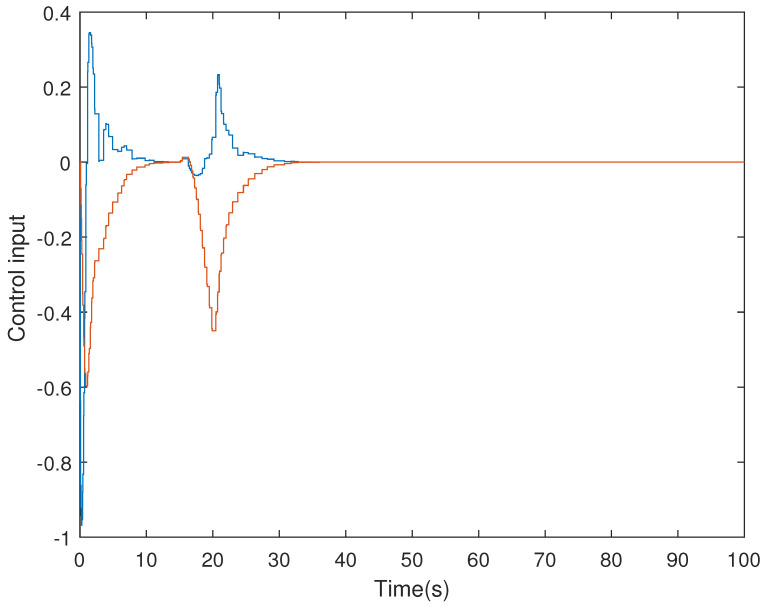
Control input of the LFC system based on the adaptive ETS in Case 2.

**Figure 8:**
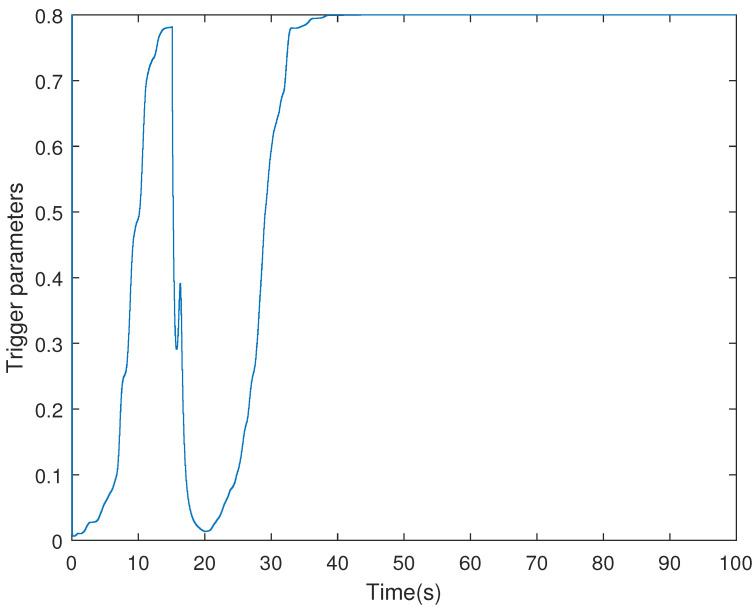
The threshold σ(t) of the system with the adaptive ETS in Case 2.

The simulated results of Case 2 are shown in Figure 6, Figure 7 and Figure 8. Figure 6 depicts the system state trajectories, from which one can see that the state response curves of the turbine output power $\Delta H_{m}$ and frequency deviation Δa of the closed-loop system subjected to changes in load demand. Compared to Figure 2 in Case 1, the turbine output power $\Delta H_{m}$ and the system frequency deviation Δa approach zero in a shorter time, which indicates the use of controller in Case 2 can better mitigate the impact of deception attacks and suppress the fluctuations in system frequency and restore the stability of the system. The control input of the LFC system based on adaptive ETS are displayed in Figure 7.

Figure 8 exhibits the threshold σ(t) of the system with adaptive ETS, where the triggering threshold is automatically adjusted even if the system suffers from the disturbance. When the system is stable, the adaptive threshold converges to a constant.

To reflect the merits of the proposed method in saving the network bandwidth, we compare the adaptive ETS with the conventional ETS as follows:

(i) Consider σ(t) in adaptive ETS (6) with the parameters α=0.8,ι=1.
(ii) The ETS in (6) with a fixed threshold $\bar{\sigma }$ is considered, which is reduced to a conventional ETS. Without loss of generality, the threshold is selected to be an average value that can be calculated by
  (29) $$\begin{array}{c} \bar{\sigma }=\frac{\sum_{\nu =0}^{NDS}\sigma_{\nu }}{NDS}, \end{array}$$
  where ν∈N, $\sigma_{\nu }$ denotes the ν-th the triggering threshold in adaptive ETS (6) at the ν-th sampling instant, and NDS is the number of data samplings.

Using LMIs, one can obtain the controller gains of two ETSs, which are listed in Table 3. The event-triggered constant $\bar{\sigma }=0.7$ is calculated by (29) within 60 s.

**Figure 9:**
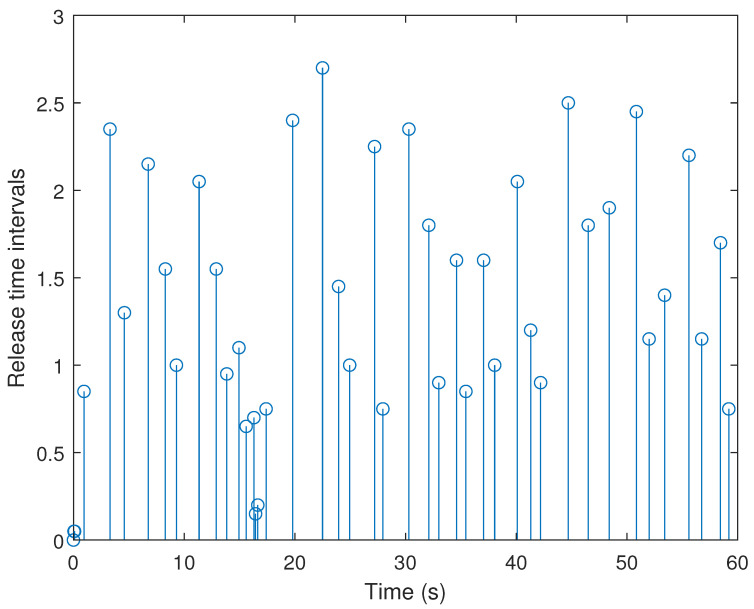
Release instants and release intervals with $\bar{\sigma }$ = 0.7.

**Figure 10:**
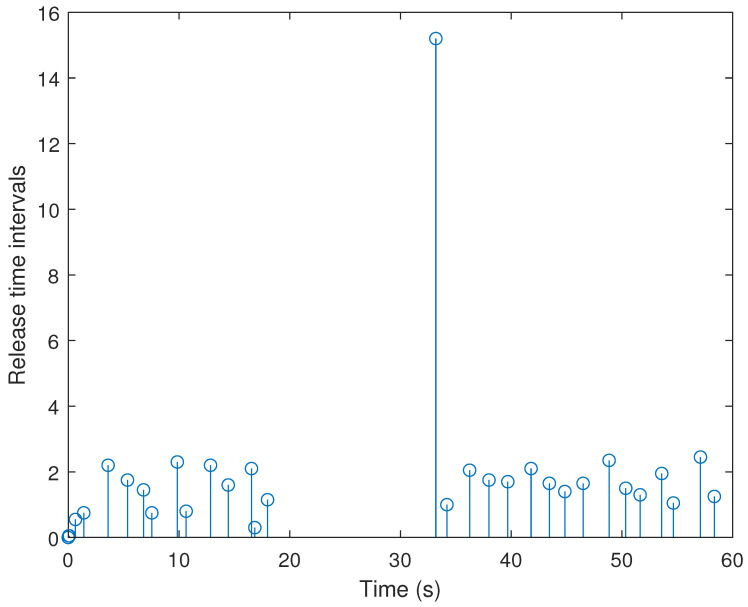
Release instants and release intervals with the adaptive ETS.

Figure 9 and Figure 10 plot the triggering and releasing intervals of the discussed system under two schemes, in which fewer sampling packets are released over the network under the adaptive ETS. For better analysis, the statistical results of the NDS, and the packet-releasing (NPR) and data-releasing rate (DRR) for two ETSs are written in Table 4, wherein $DRR=\frac{NPR}{NDS}$.

As shown in Table 4, the number of sampling data is 1200 within 60 s. Under the adaptive ETS (6) proposed in this paper, the number of released packets is 31, and the DRR is 2.58%. Compared to the DRR = 3.58% of the conventional ETS, the proposed adaptive ETS can significantly reduce the transmission of unnecessary packets. This indicates that more communication resources can be saved by utilizing our developed adaptive ETS.

## 5. Conclusions

The problem of $H_{\infty }$ LFC has been addressed for LFC systems under deception attacks by applying the developed adaptive ETS in this paper. To solve the issues of limited communication resources and deception attacks, a new adaptive ETS has been proposed, by which the thresholds for event-triggered conditions could be adapted to the changes in the system state under deception attacks. Based on the adaptive ETS, the average data released are lower than under the conventional ETS, and the perfomance of LFC systems subject to deception attacks can be guaranteed. Finally, the simulation results demonstrate the reliability of our proposed scheme. In future research, actuator saturation for LFC systems under cyber attacks will be worth consideration under the proposed adaptive ETS.

## Figures and Tables

**Table 1:** Meanings of the symbols for the LFC system.

Symbol	Meaning
$T_{g}$	Time constant of governor
$\Delta H_{m}\left(s\right)$	Mechanical output of the generator
$\Delta H_{d}\left(s\right)$	External interference
u(s)	Control output
ACE(s)	Area control error
E	Generator damping coefficient
M	Moment of inertia of the generator
Δa(s)	Frequency deviation
μ	Frequency bias factor
J	Speed drop
$T_{ch}$	Time constant of turbine
$\Delta H_{v}\left(s\right)$	Position deviation of the valve

**Table 2:** System parameters utilized in simulintion section.

Physical Quantity	M (kg·m${}^{2}$)	J (Hz p.u. MW${}^{-1}$)	$T_{g}$ (s)	$T_{\mathrm{ch}}$ (s)	μ	E
Values	0.1667	2.4	0.08	0.3	0.425	0.0083

**Table 3:** Controller gains of two ETSs.

Schemes	Controller Gains K
General ETS with fixed threshold ($\bar{\sigma }$ = 0.7)	[0.0393 0.5584]
This work	[0.0374 0.5270]

**Table 4:** The number of packets transmitted in 60 s with sampling period h=0.05.

Schemes	NDS	NPR	DRR
General ETS with fixed threshold ($\bar{\sigma }$ = 0.7)	1200	43	3.58%
This work	1200	31	2.58%

## Data Availability

Not applicable.
